# Characterization of Lung Fibroblasts More than Two Decades after Mustard Gas Exposure

**DOI:** 10.1371/journal.pone.0145148

**Published:** 2015-12-17

**Authors:** Gila Pirzad Jahromi, Mostafa Ghanei, Seyed Kazem Hosseini, Alireza Shamsaei, Mazaher Gholipourmalekabadi, Ameneh Koochaki, Nushin Karkuki Osguei, Ali Samadikuchaksaraei

**Affiliations:** 1 Neuroscience Research Centre, Baqiyatallah University of Medical Sciences, Tehran, Iran; 2 Chemical Injury Research Centre, Baqiyatallah University of Medical Sciences, Tehran, Iran; 3 Department of Respiratory Medicine, Faculty of Medicine, Baqiyatallah University of Medical Sciences, Tehran, Iran; 4 Tissue Bank & Preparation Research Centre, Tehran University of Medical Sciences, Tehran, Iran; 5 Department of Pathology, Faculty of Medicine, Baqiyatallah University of Medical Sciences, Tehran, Iran; 6 Biotechnology Department, School of Advanced Technologies in Medicine, Shahid Beheshti University of Medical Sciences, Tehran, Iran; 7 Cellular and Molecular Biology Research Center, Shahid Beheshti University of Medical Sciences, Tehran, Iran; 8 Eposcience Millennium Institute, Tehran, Iran; 9 Cellular and Molecular Research Centre, Iran University of Medical Sciences, Tehran, Iran; 10 Department of Tissue Engineering and Regenerative Medicine, Faculty of Advanced Technologies in Medicine, Iran University of Medical Sciences, Tehran, Iran; 11 Department of Medical Biotechnology, Faculty of Allied Medicine, Iran University of Medical Sciences, Tehran, Iran; Medical University of South Carolina, UNITED STATES

## Abstract

**Purpose:**

In patients with short-term exposure to the sulfur mustard gas, the delayed cellular effects on lungs have not been well understood yet. The lung pathology shows a dominant feature consistent with obliterative bronchiolitis, in which fibroblasts play a central role. This study aims to characterize alterations to lung fibroblasts, at the cellular level, in patients with delayed respiratory complications after short-term exposure to the sulfur mustard gas.

**Methods:**

Fibroblasts were isolated from the transbronchial biopsies of patients with documented history of exposure to single high-dose sulfur mustard during 1985–7 and compared with the fibroblasts of control subjects.

**Results:**

Compared with controls, patients’ fibroblasts were thinner and shorter, and showed a higher population doubling level, migration capacity and number of filopodia. Sulfur mustard decreased the *in vitro* viability of fibroblasts and increased their sensitivity to induction of apoptosis, but did not change the rate of spontaneous apoptosis. In addition, higher expression of alpha smooth muscle actin showed that the lung's microenvironment in these patients is permissive for myofibroblastic differentiation.

**Conclusions:**

These findings suggest that in patients under the study, the delayed pulmonary complications of sulfur mustard should be considered as a unique pathology, which might need a specific management by manipulation of cellular components.

## Introduction

Sulfur mustard (SM) is a chemical warfare agent that can damage multiple organs. The damage is due to a combination of mechanisms involving DNA alkylation and alteration of several signaling pathways [1, 2]. In the respiratory system, the acute toxic effects include pseudomembrane formation or airway obstruction from fibrin/necrotic epithelial cell sloughing, which is a great contributor of death from acute inhalation-related SM toxicity [3, 4]. Acute toxic effects are manifested in a dose-dependent manner as laryngitis and tracheobronchitis [5], airway epithelial cell death [6, 7] and respiratory distress syndrome [5]. The delayed long-term pathological changes observed after examination of lung biopsies are reported to be similar to chronic bronchiolitis [8, 9], parenchymal lung fibrosis and obliterative bronchiolitis [10–14]. But, the collective histopathological lung parenchymal and airway epithelial changes cannot be exactly defined by any of these three histopathological conditions.

Although many studies have been conducted to determine the cellular and molecular mechanisms of acute SM-induced injuries in different organs [2, 15], little has been done to understand the mechanisms governing the delayed pathological changes in the respiratory system. The importance of these changes lies in the fact that respiratory complications are the most common long-term medical problems reported, more than 20 years after initial exposure to sulfur mustard, among 34,000 Iranian war survivors [16]. These affected individuals face a progressive time-dependent process i.e. pulmonary pathology worsens with passage of time [17]. The patients would benefit from any intervention that can reverse or stop the progression of histological changes [18]. Therefore, more in-depth studies are needed to elucidate the underlying pathogenic mechanisms and define new targets for therapeutic interventions.

As pulmonary fibroblasts and the epithelial cells of airways and alveoli are involved in lung pathology after exposure to SM, isolation of these cells directly from the affected individuals for *in vitro* studies of their behavior could be used, in order to elucidate the mechanism(s) leading to their altered function. Fibroblasts are the major players in most fibrotic diseases. Also, alteration in the fibroblasts phenotype and function has been reported in a variety of other lung diseases such as asthma [19], chronic obstructive pulmonary disease [20], and pulmonary hypertension [21]. Here, we report isolation of primary lung fibroblasts from patients with delayed respiratory complications of SM and normal controls and show the phenotypic characterization of these fibroblasts in terms of morphology, proliferation, migration, apoptosis and the expression of myofibroblastic markers.

## Materials and Methods

### Patients and samples

Biopsies were obtained in Baqiyatallah Hospital as the main referral Centre for the chemically-injured patients in Tehran. The study has been approved by Baqiyatallah Hospital’s Ethical Committee and written informed consent was obtained from all the participants. Five patients with documented chemical injury with SM after twenty years of exposure and documented delayed pulmonary complications were entered into this study. The documentation of SM exposure was based on official certification issued by the Iranian Veterans Foundation, which is the official center for compensation of war-disabled victims. Patients with a history of smoking and occupational exposure to toxic agents and having dusty jobs were excluded from the study. Normal tissues of the lungs of patients who underwent lobectomy/pneumonectomy for removal of a primary lung tumor were used as the control. Characteristics of the patients, control subjects and the patients’ inclusion and exclusion criteria are shown in Tables 1 and 2.

**Table 1:** Characteristics of the subjects and biopsy specimens.

	SM-exposed patients	Controls
Subjects	4 males and 1 female	4 males
Mean age (years)	55	57
RV (% predicted)	160.3 ± 62.5	105.9 ± 9.6
FVC (% predicted)	66.1 ± 13.2*	88.2 ± 5.9
FEV1 (% predicted)	58.2 ± 17.6**	90.7 ± 6.5
FEV1/FVC (%)	65.4 ± 8.7***	88 ± 5.8
Intervention	Transbronchial biopsy	Lobectomy / pneumonectomy for removal of a primary lung tumour
Biopsy specimen	Lung parenchyma	Normal lung parenchyma

Abbreviation: SM sulfur mustard

Values are shown as mean ± SEM

*p <0 .05

**p <0 .01

***p <0 .001

**Table 2:** Patients inclusion and exclusion criteria.

Inclusion criteria	Exclusion criteria
Official documentation of exposure to SM by the Iranian Veteran Foundation	History of smoking
Exposure to a single high-dose of SM	Occupational exposure to toxic inhalants and dusts
Exposure during 1985–1987 (during Iran-Iraq war)	
Suffering from persistent respiratory and chest discomfort, shortness of breath and cough	
Exercise intolerance	

Abbreviation: SM sulfur mustard

SM-exposed patients underwent bronchoscopic lung biopsy with a flexible fiber-optic bronchoscope (Olympus BFIT, Tokyo, Japan). For this purpose, the upper respiratory tract was locally anesthetized with 2% lidocaine solution and 0.75 mg atropine was administered intramuscularly. The bronchoscope was introduced into the bronchial tree and three to four bronchial biopsies were taken from locations in the distal trachea and main-stem bronchi using FB-15C-Olympus forceps. The samples from each patient were pooled for cell isolation and culture.

### Cell isolation and culture

Fibroblasts were isolated according to the previously published methods [22–24]. Lung specimens were immediately washed with PBS and transferred to Hank’s Balanced Salt Solution (HBSS) supplemented with 50 U/ml penicillin and 50 μg/ml streptomycin (all from Gibco Invitrogen, Germany) at 4°C. Upon arrival to the cell culture room, the specimens were minced into pieces smaller than 1 mm^3^ and placed in 6-well culture plates (3 pieces per well) with a thin layer of fetal bovine serum (FBS). The pieces were left for 10 minutes in incubator to let them attach to the culture surface. Then, 1.5 ml of RPMI 1640 medium (Gibco, Germany), supplemented with 10% fetal bovine serum (FBS) (Gibco, Germany), 50 U/ml penicillin (Sigma, Germany), 50 μg/ml streptomycin, 0.25 μg/ml amphotericin B (Invitrogen, Germany), 8 mM L-glutamine (Sigma, Germany), and 1% (v/v) MEM Vitamins Solution (100×) (Gibco Invitrogen, Germany) was added to the wells. All these and subsequent cultures were maintained in an incubator at 37°C in a humidified atmosphere of 5% CO2/95% air. Culture medium was changed 2 days later, and three times a week thereafter. The explanted cells were sub-confluent after about 2–3 weeks, when they were trypsinized and subcultured.

### Immunocytochemistry

Cells of passage four [25] were seeded onto the microscope slides and cultured overnight. Then, they were fixed for 2 minutes in the ice-cold 100% acetone (Merck, Germany) and their endogenous peroxidase activity was blocked by immersion of samples in a 0.3% solution of H_2_O_2_. Afterwards, the samples were incubated at 4°C overnight with primary antibodies including mouse polyclonal anti-vimentin (1/100 dilution), mouse monoclonal anti-pancytokeratin (1/100 dilution), mouse monoclonal anti-α-SMA (1/100 dilution) (all from Dako, Germany) and mouse monoclonal anti-fibronectin (1/100 dilution) (Sigma, Germany). The cells stained without application of primary antibodies served as controls. The day after, the antibody binding sites were visualized by incubation of samples in biotinylated rabbit anti-mouse secondary antibodies (1/250 dilution) (Abcam, Germany) followed by application of Biotin-Streptavidin (ABC) IHC detection kit (Abcam Inc., Cambridge, MA, USA) and diaminobenzidine tetrahydrochloride (DAB) (Sigma-Aldrich, Germany). All the samples were counter-stained with Hematoxylin. Images were captured using Infinity Capture Imaging Software (Lumenera Corporation, Ottawa, ON, Canada).

Where appropriate, cell counting was performed by counting 100 nucleated cells in four corners and center of the slide (40 cells at each area) and positively stained cells were identified.

### Morphological characterization

Crystal Violet staining was used for morphological assessment. For this purpose, the cells were cultured in four-well chamber slides (10^5^ cells/well) overnight. They were fixed next day with 1% glutaraldehyde for 30 minutes, stained for two hours [26] in 0.5% Crystal Violet and viewed under a light microscope at 100× [27]. Under the light microscope, each well was started to be viewed from the left upper corner and the first 5 cells that were not in contact with other cells and were distinctly visible were identified (sum of 20 cells for a 4-well chamber slide). The length and width of each cell was measured using the eyepiece reticle and their dimensions were recorded as described by Larsen *et al*, 2004 [27].

### Transmission electron microscopy

Transmission electron microscopy (TEM) was performed in order to detect filopodia. For this purpose, the cells were trypsinized, washed with PBS, fixed in 2.5% glutaraldehyde in 0.1 M phosphate buffer, post-fixed in 1% osmium tetroxide and finally embedded in epoxy resin. Thin sections of 60 nm were stained with uranyl acetate followed by lead citrate and examined with a ZIESS electron microscope (EM906A) at 80KV as previously described [28, 29].

### Population doubling level

At passage two [24], cells from three randomly selected subjects of each group were seeded at 10^5^ cells per 25 cm^2^, cultured until sub-confluent for 7 days with regular changes of medium, harvested by treatment with trypsin / EDTA, counted and transferred to the next passage. Population doubling levels were determined at the time of passaging and weekly passaging was continued (on days 7, 14, 21, 28 and 35) until the harvested cell numbers dropped below the initial seeding number of 10^5^ on the day 35. The population doubling level (PDL) was calculated as described by McAteer and Davis 2002 [30]:

$$PDL=3.32(\text{log}\;N_{H}-\text{log}\;N_{I})$$

where N_H_ and N_I_ are the number of the harvested cells and the inoculated cells, respectively. The data were summarized in a column chart and each individual culture's PDL was presented in accordance with the report by Mio and co-workers [22].

### Migration assay

The migration assay was performed as described by Larsen *et al*, 2006 [31] and Scheja *et al*, 2007 [32]. A cloning cylinder was used to delineate seeding of 30,000 fibroblasts. The cells were allowed to seed for 6 hours, then the cylinder was removed and the cells were allowed to migrate for an additional 48 hours. The cells were fixed with 1% glutaraldehyde for 30 minutes, stained with Crystal Violet and viewed under the light microscope as described above. Then, the distances of 200 cells from the border of the removed cylinder were measured using the eyepiece reticle.

### Induction of apoptosis

In order to assess the response of the cells to triggers of apoptosis, passage-four fibroblasts were treated with various concentrations of H_2_O_2_ (500, 700 and 900 μM) for 4 hours [33]. Apoptosis and necrosis were assessed by flow cytometry of fibroblasts at the mid-log phase of their growth after staining with ApopNexin FITC Apoptosis Detection kit in combination with propidium iodide (PI) (Chemicon; Germany). In this kit, the annexin V conjugated to green fluorescent FITC dye detects phosphatidylserine on the surface of apoptotic cells and PI, the red fluorescent dye, permeates into the dead cells. After staining, the apoptotic cells show a green fluorescence, dead cells show red and green fluorescence and live cells show little or no fluorescence. Flow cytometry reading was taken using 488 nm excitation and band pass filters of 530 nm (for FITC detection) and 585 nm (for PI detection).

### Statistical analysis

For statistical analysis, the results were expressed as means ± SEM. Statistical analyses were made using Student’s t test and ANOVA for comparison of two and several means, respectively. Also, comparison of proportions were made using chi square test. Values of < 0.05 were considered statistically significant.

## Results

### Immunocytochemistry

**Fig 1:**
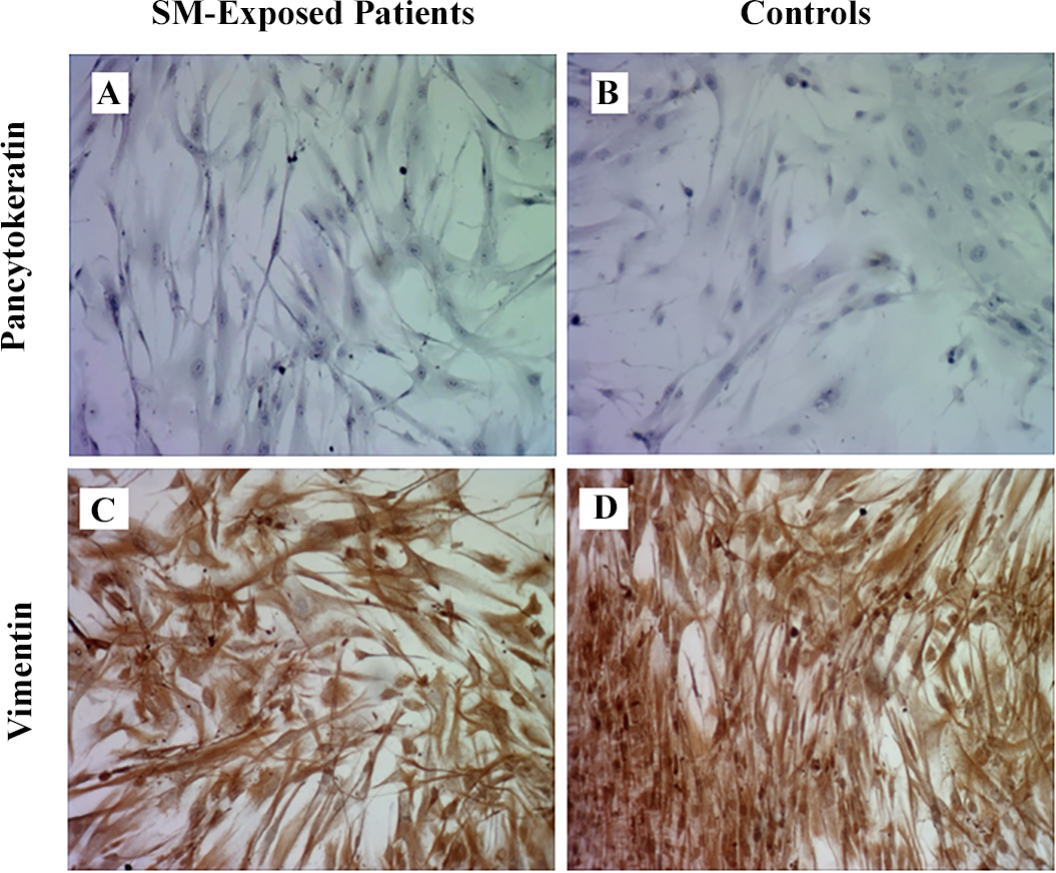
Immunocytochemical characterization of fibroblasts. The cells from both SM-exposed patients and controls were negative for pancytokeratin staining (A and B; 10×) and positive for vimentin (C and D; 10×).

All the cells were negative for pancytokeratin (Fig 1A and 1B), an epithelial marker, and 94.78% of the cells were positive for vimentin, the most frequently found intermediate filament in fibroblasts (Fig 1C and 1D). Under the light microscope, the cells showed a fibroblast-like spindle-shaped morphology. No cell with epithelial-like morphology was detected. A combination of spindle-shaped morphology, positive staining for vimentin and negative staining for the epithelial marker confirms the fibroblast phenotype of the cells under the study [34].

**Fig 2:**
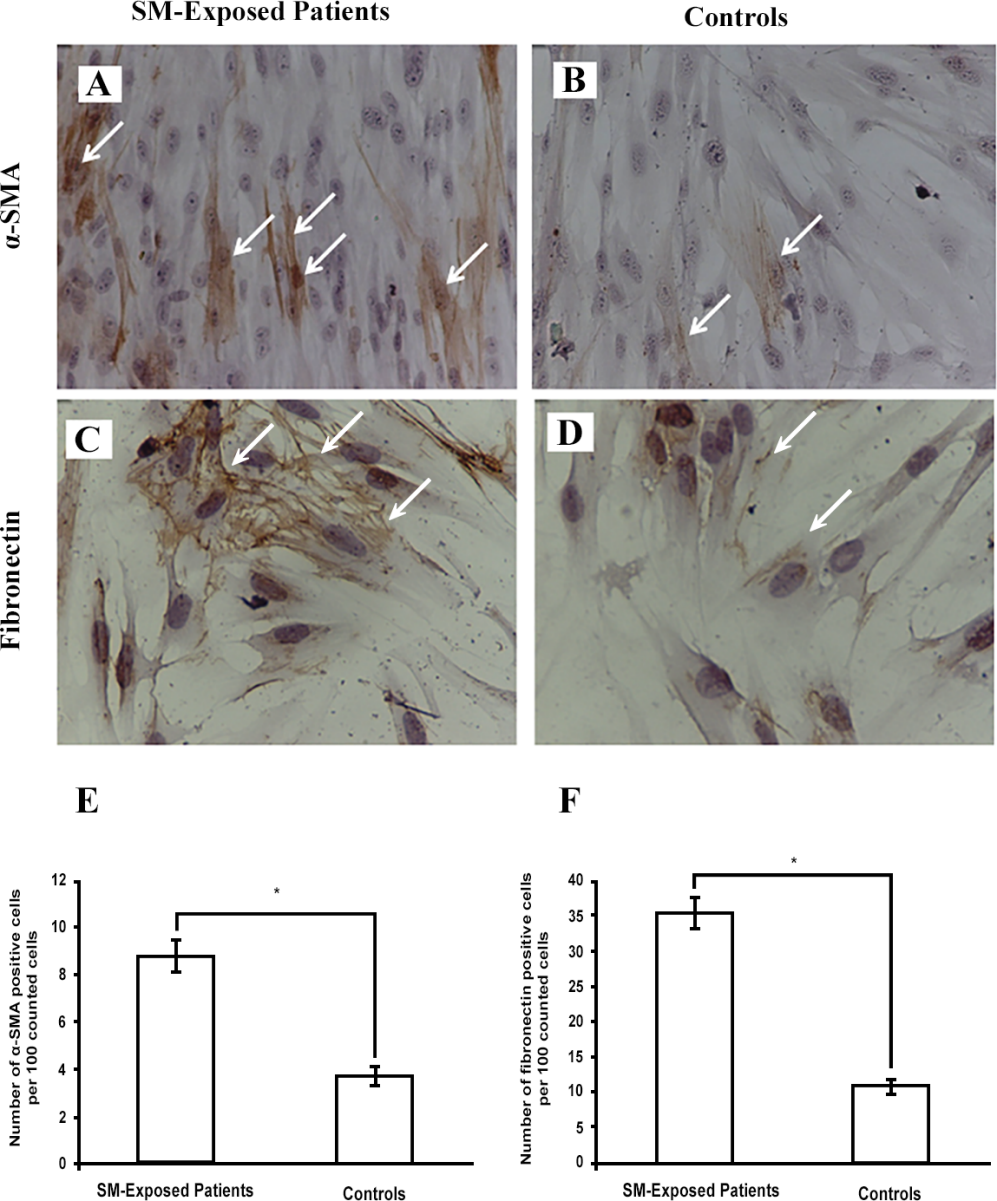
Immunocytochemical detection of α-SMA and fibronectin expression. Both SM-exposed patients and controls expressed α-SMA (A and B, 10×) and fibronectin (C and D, 20×). The number of positive cells was determined by enumeration of 100 cells/sample. Figures E and F present the count of positive cells as mean ± SEM for patients (n = 5) and controls (n = 4). *p < 0.0001. Arrows show positive cells; Abbreviations: α-SMA alpha smooth muscle actin, SM sulfur mustard.

Immunocytochemical staining for the myofibroblast marker alpha smooth muscle actin (α-SMA) (Fig 2A and 2B) showed that the expression of this marker in 100 cells counted in SM-exposed patients was 2.32 fold higher than 100 cells counted in controls (p ≤ 0.0001) (Fig 2E). Also, the fibronectin staining (Fig 2C and 2D) showed that in 100 counted fibroblasts, those of SM-exposed patients express this glycoprotein 3.31 fold higher than controls (p ≤ 0.0001) (Fig 2F).

### Morphological characterization

**Fig 3:**
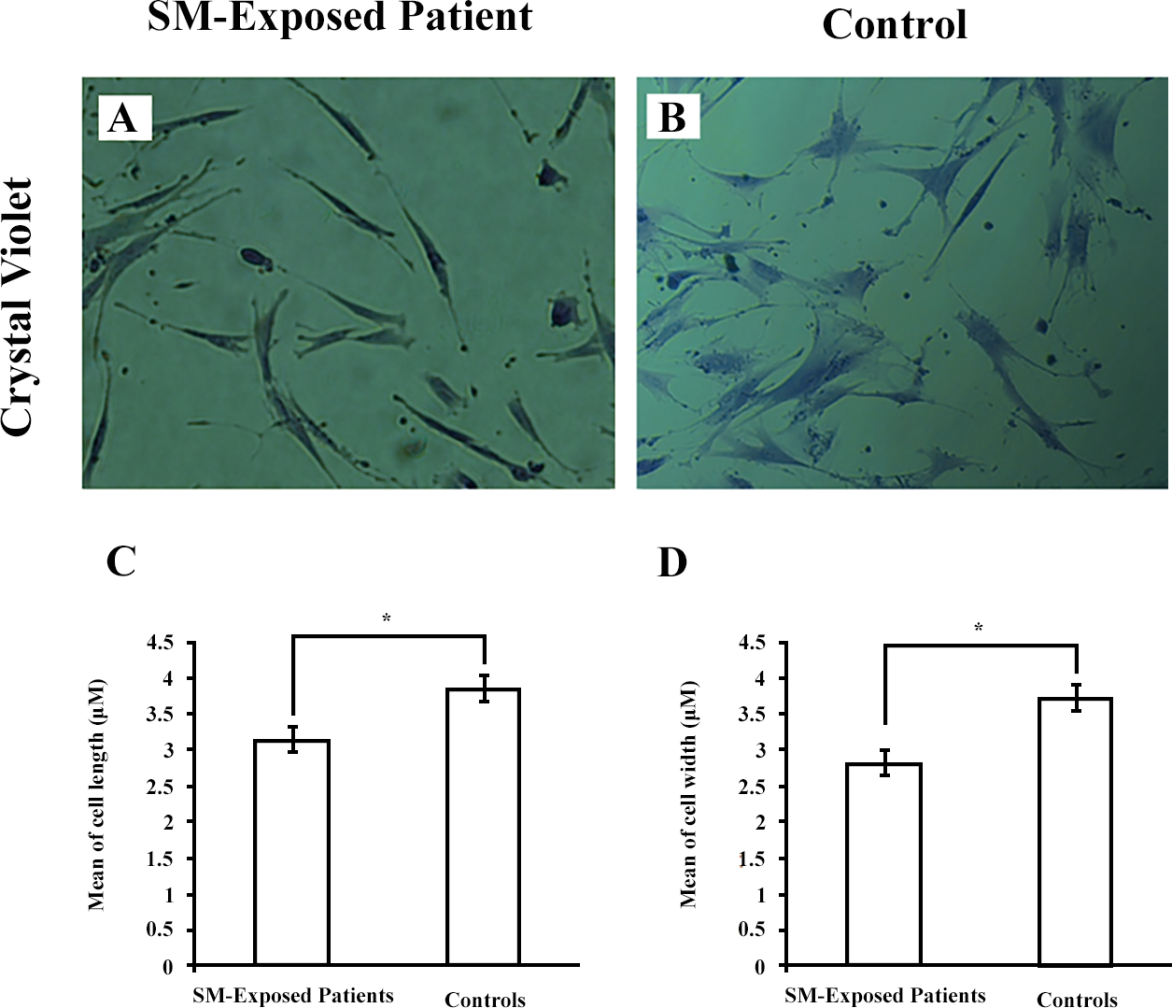
Morphologic characterization of fibroblast. Fibroblasts from both SM-exposed patients and controls (A and B; 40×) were stained with Crystal Violet. Twenty cells/sample were measured for each patient. The results are presented for both length (C) and width (D) as mean ± SEM for patients (n = 5) and controls (n = 4). *p < 0.05. Abbreviations: SM sulfur mustard.

Compared with fibroblasts from controls (Fig 3B), fibroblasts derived from SM-exposed patients displayed significantly shorter and thinner morphologies (Fig 3A and 3B). These morphologies were stably observed in all passages (data not shown). The measurements made using the eyepiece reticle revealed that mean (±SEM) length of fibroblasts in SM-exposed patients was 3.16 ± 0.189 μm and that in controls was 3.87 ± 189 μm (p < 0.05) (Fig 3C). Also, the mean of width of fibroblasts was 2.86 ± 0.133 μm in SM-exposed patients and 3.76 ± 0.145 μm in controls (p < 0.01) (Fig 3D).

### Transmission electron microscopy

**Fig 4:**
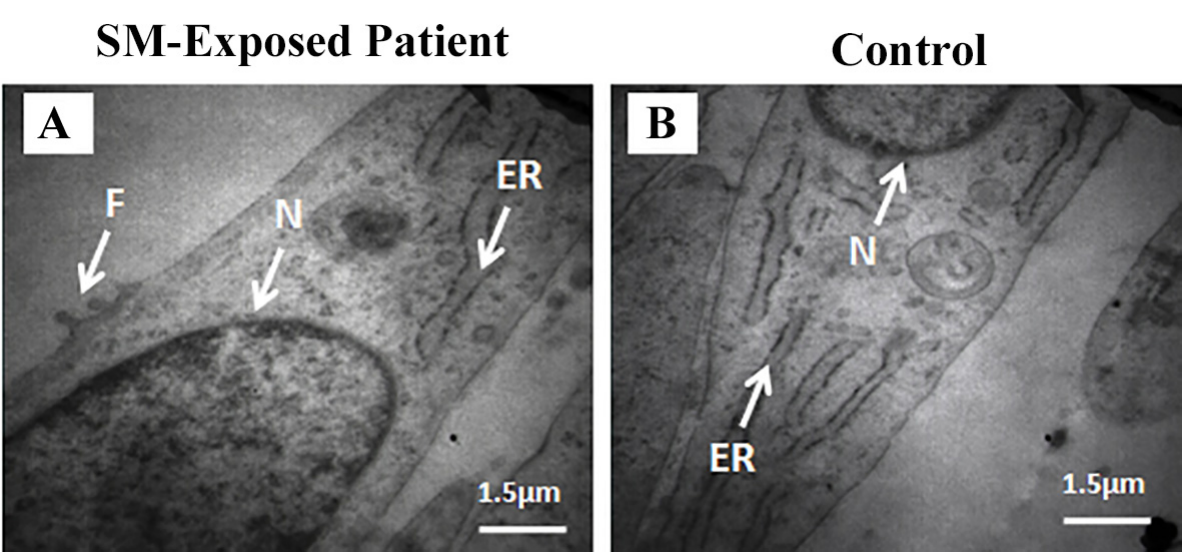
Electron microscopic photomicrographs of fibroblasts. SM-exposed patient and control (A and B). Filopodia are seen in SM-exposed fibroblasts. Abbreviations: SM sulfur mustard, ER endoplasmic reticulum, F filopodium, N nucleus.

According to the previous report of morphology of filopodium in lower respiratory tract fibroblasts [27], the electron microscopic study revealed filopodia in the SM-exposed patients' fibroblasts. But, no filopodium was seen in control fibroblasts (Fig 4A and 4B). As filopodia have been highly implicated in cell migration [35], this finding suggests that the migratory capacity of SM-exposed fibroblasts is higher than controls.

### Population doubling level

**Fig 5:**
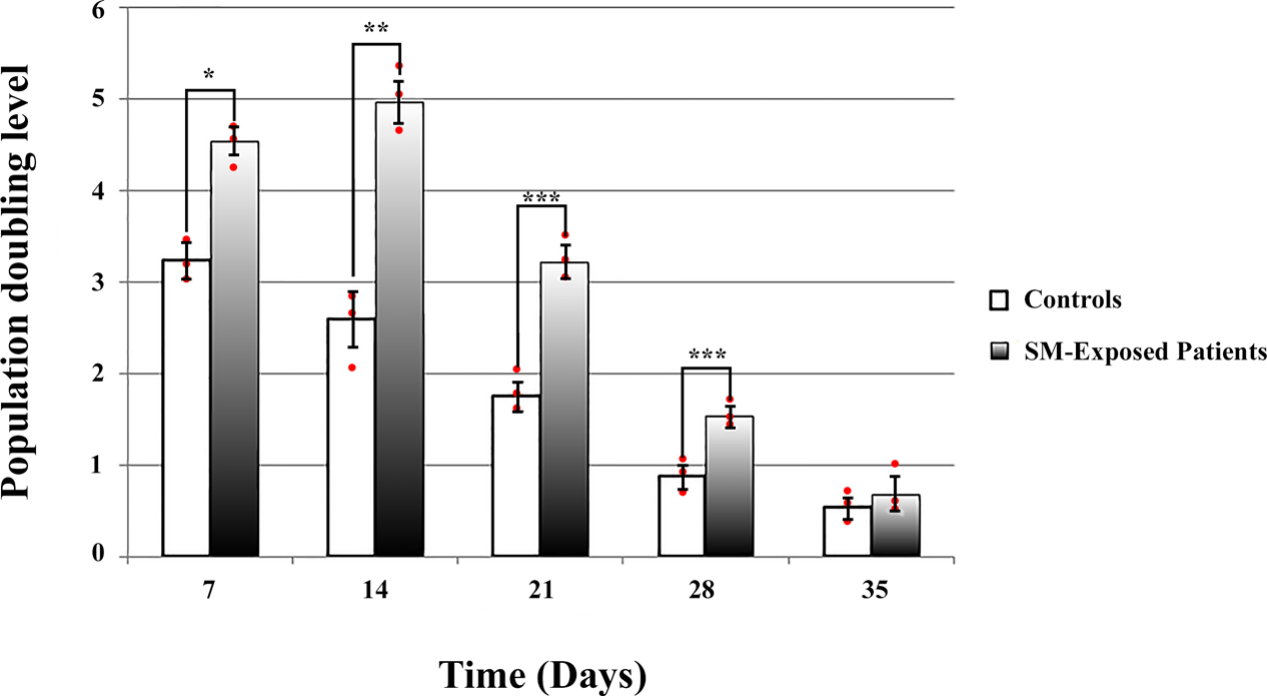
Population doubling levels (PDLs). PDLs of SM-exposed patients and controls were compared at each passage performed at different time points after the start of the primary culture. Each individual culture's PDL was presented by a red dot. PDL of the SM-exposed patients were significantly higher than controls from the day 7 through 28, but did not show any significant difference on the day 35. * p = 0.0025, ** p = 0.0001, *** p < 0.05.

Calculation of the population doubling level (PDL) of fibroblasts showed a significantly higher level in chemically injured patients (n = 3) compared with controls (n = 3) on days 7 (p = 0.0025), 14 (p = 0.0001), 21 (p = 0.037), and 28 (p = 0.0147) (passages 1 through 4). However, a progressive decrease in PDL was observed due to *in vitro* cellular senescence until, at passage 5 (day 35), it approached the same level in both patients and controls (Fig 5). Statistical comparisons were made using Student's t test.

### Migration assay

**Fig 6:**
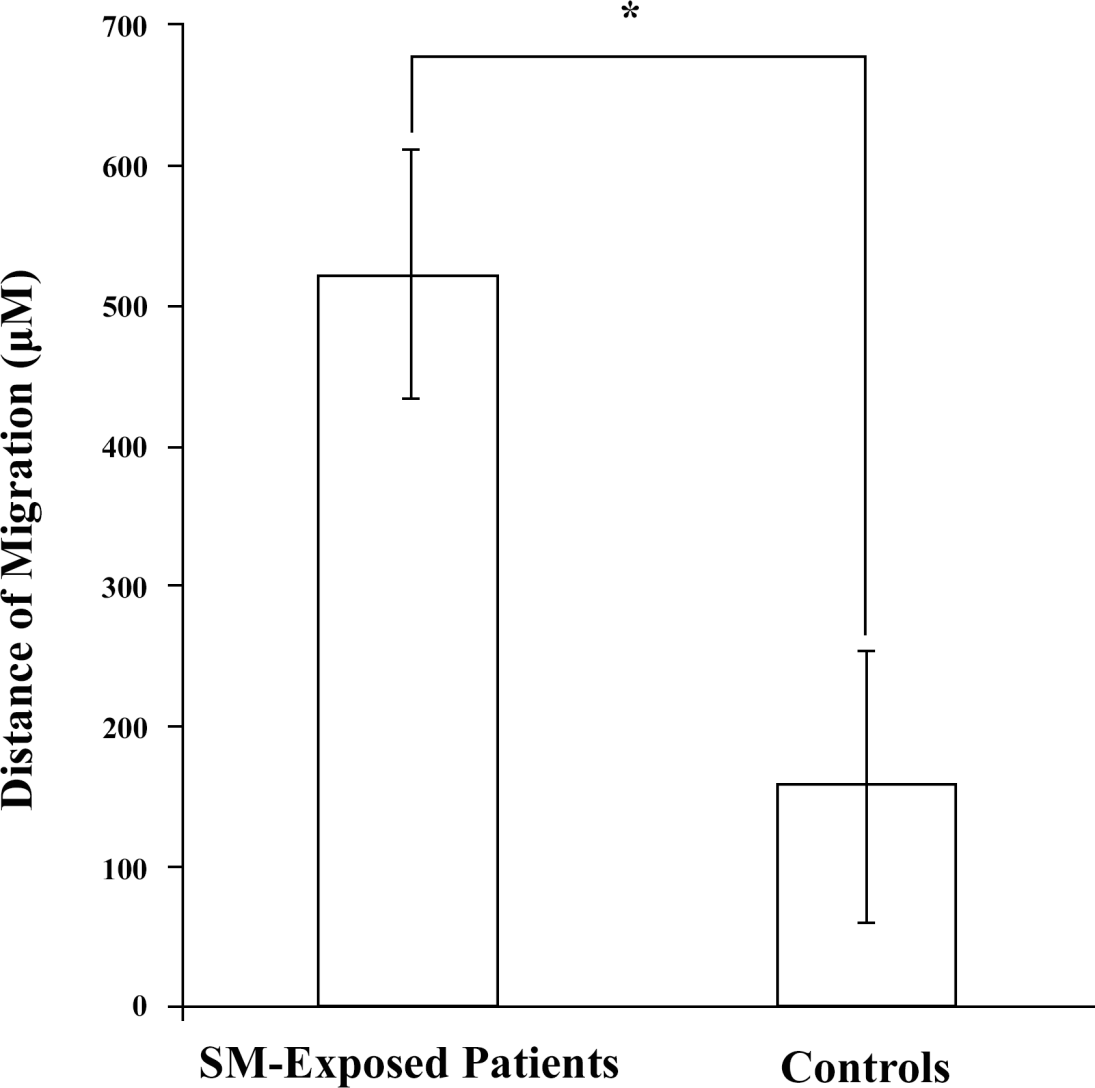
Cell migration. The migration distance of fibroblasts isolated from SM-exposed patients (n = 5) was significantly higher than the distance migrated by controls (n = 4); *p < 0.05. Abbreviations: SM sulfur mustard.

The migration assay showed that after 48 hours, the distance of the fibroblasts from the SM-exposed patients was three-folds longer than those from the controls (p = 0.025) (Fig 6). The migration distance measured for control fibroblasts conforms to the control values reported by Bermudez *et al*, 2013 [36] confirming the accuracy of the measurements.

### Induction of apoptosis

**Fig 7:**
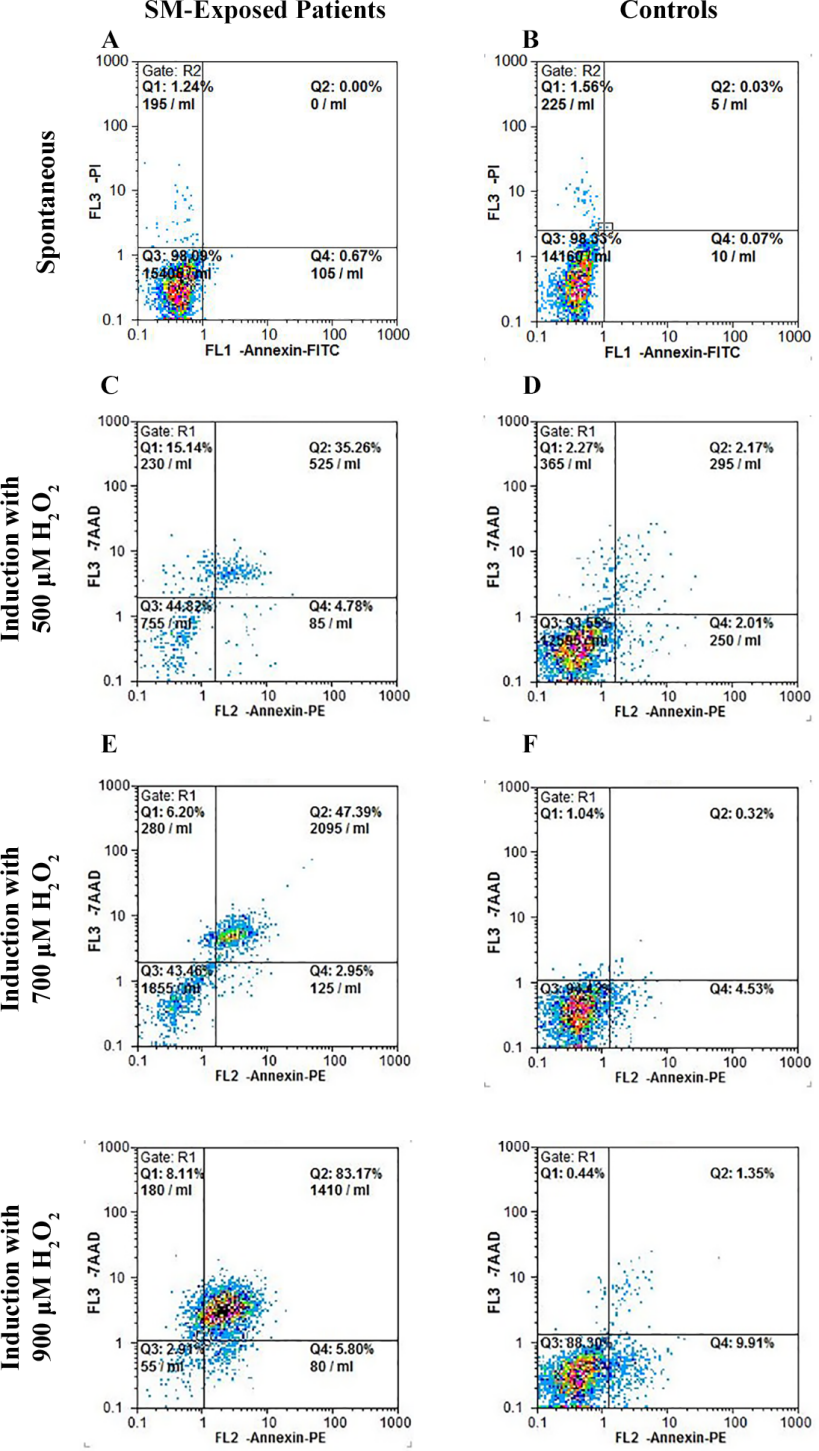
Flow cytometric analysis for apoptosis of fibroblasts. Representative dot plots of the flow cytometric analysis of the Annexin V–FITC/propidium iodide (PI) following induction of apoptosis with H_2_O_2_. Fibroblasts derived from SM-exposed patients and controls were assessed for spontaneous apoptosis and apoptosis following treatment with 500, 700 and 900 μM of H_2_O_2_. Spontaneous apoptosis was not significantly different in SM-exposed patients and controls. However, a higher dose-dependent sensitivity to H_2_O_2_ is observed in patients as compared with controls. Q_1_: necrotic cells, Q_2_: late apoptotic cells, Q_3_: viable cells, and Q_4_: early apoptotic cells.

As shown in Fig 7, the results of flow cytometric analysis for apoptosis/necrosis revealed that the SM-exposed patients and controls did not differ significantly in the percentage of spontaneous cellular apoptosis (Fig 7A and 7B). But, in comparison with the controls, the cells derived from the SM-exposed patients showed a high sensitivity to induction of apoptosis by H_2_O_2_ in a dose-dependent manner (p < 0.05; chi square). On the other hand, the cells derived from the SM-exposed patients had a higher percentage of necrosis.

## Discussion

We have isolated parenchymal lung fibroblasts from the patients suffering from the long-term respiratory complications of SM and determined their cellular characteristics. The fibroblastic identity of the isolated cells were confirmed by their spindle-shaped morphology, lack of the cells with epithelial morphology under the light microscopy, positive staining for vimentin and negative staining for the epithelial marker pancytokeratin [34]. Compared with controls, these fibroblasts were shorter and thinner, showed filopodial protrusions, had higher population doubling level and migration capacity, and were more sensitive to induction of apoptosis by H_2_O_2_. The high expression of myofibroblastic marker α-SMA was another interesting finding in this study. The myofibroblasts contribute to the synthesis of extracellular matrix and formation of the fibrotic conditions [37]. Therefore, modulation of differentiation into myofibroblasts and modification of their function could be considered as a point of intervention for management of delayed complications after acute exposure to SM.

Several reports of the pathological examination of lung tissue in SM-exposed patients, who suffered from the delayed respiratory complications, identified a collective parenchymal and airway epithelial changes, which conformed to two pathologies of obliterative bronchiolitis (OB) and parenchymal lung fibrosis [9–12]. The former has been shown to be the major feature not only in patients underwent open lung biopsy, but also in those who were studied with high-resolution CT scanning and broncho-alveoar lavage (BAL) [12].

OB is a pathological feature observed in various conditions such as chronic lung allograft rejection, post-viral infections (such as adenovirus and measles), allogeneic hematopoietic stem cell transplantation or exposure to inhalation toxins [38]. In a recent report on patients with OB due to chronic lung allograft rejection, the cellular characteristics of parenchymal airway fibroblasts, isolated 6 and 12 months after lung transplantation, have been studied [39]. It has been shown that the proliferation rate and migratory capacity of these fibroblasts were decreased in this condition. These finding are in contrast with what we found in current study. Here, we have shown that the population doubling level and migratory capacity of lung parenchymal fibroblasts, isolated more than 20 years after exposure to SM, were increased. Also, we identified filopodial protrusions in the fibroblasts derived from the SM-exposed patients, which is another indicator of increased migratory capacity.

Parenchymal lung fibrosis is observed in various conditions including idiopathic pulmonary fibrosis (IPF). In IPF varying results have been reported in regards to the proliferation rate of lung parenchymal fibroblasts isolated 22 ± 6 months after the first visit after the beginning of the symptoms. The results showed both increase [23] and decrease [40] of proliferation, which were possibly linked to the initiating pathogenic mechanism. Also, equal rates of spontaneous cellular apoptosis that we observed in fibroblasts of SM-exposed patients and controls is in contrast to several reports of reduced apoptosis observed in idiopathic pulmonary fibrosis [41]. Although, the time of biopsy taken after sustaining the injury widely varies in the current study and the above studies on OB and idiopathic pulmonary fibrosis, the behavior of fibroblasts could be compared as the biopsies are taken from the lungs at the stage of established disease. The differences of the phenotype of fibroblasts in SM-exposed patients and patients with other conditions might be explained by development of a sulfur mustard-specific microenvironment after exposure and its preservation in subsequent years.

Here, we report that patients fibroblasts are thinner and shorter than controls. This morphologic difference may implicate that resident lung fibroblasts are not the source of these cells. The higher migratory capacity of these cells may also indicate that the cells have been attracted to the site of pathology and implicate their non-residential source. Determination of the origin of these fibroblasts can identify a potentially crucial point for intervention. The importance of the cell origin can be illustrated by a recent experiment by Harris *et al*, 2013 [42]. They have shown that inhibition of C-X-C motif chemokine 12 (CXCL12) (also known as the stromal cell-derived factor 1 [SDF-1]) attenuates obliterative bronchiolitis in a murine heterotopic tracheal transplant model. The attenuation was due to blocking of fibrocytes migration and differentiation as the source of fibrosing cells. As in the current animal models of chronic complications of SM exposure (mouse [43], rat and monkey [44]), assessment of the complications is only feasible in a short-term period (after 14 days of exposue), the results may not be applicable to human subjects that suffer complications more than 20 years after acute exposure. This lack of a suitable animal model of the delayed complications makes detailed study of its pathogenic mechanisms, including cell lineage tracing, very challenging. However, it should be noted that extrapulmonary origin of fibroblasts has been implied in several other chronic lung conditions such as idiopathic pulmonary fibrosis and obliterative bronchiolitis (see Lama and Phan, 2006 [45] and Andersson-Sjöland *et al*, 2011 [46] for review). The question of lineage relationship remains to be answered as a valuable step in understanding the mechanisms of this disease.

The other interesting finding in this report is the high sensitivity of SM-exposed fibroblasts to induction of apoptosis. Combination of this finding with low *in vitro* viability of SM-exposed fibroblasts, indicated by higher percentage of necrosis, and the high rate of fibroproliferation emphasizes that unique pathogenic mechanisms are involved in production and maintenance of delayed respiratory complications after SM exposure. These mechanisms should be specifically defined in order to tailor an optimized management strategy.

## Conclusion

Our study shows some of the cellular characteristics of fibroblasts isolated from patients with delayed pulmonary complications of sulfur mustard. Compared with controls, these fibroblasts show smaller dimensions, higher proliferation and migration capacities and higher sensitivity to induction of apoptosis. These findings implicate that the delayed pulmonary complications of SM should be considered as a unique pathology, which might need a specific management by manipulation of the cellular components that preserves this condition for many years.
